# Evaluation inflammatory markers of hemogram parameters in primary ovarian insufficiency

**DOI:** 10.4274/tjod.galenos.2019.09476

**Published:** 2020-04-06

**Authors:** Bülent Demir, Süreyya Sarıdaş Demir, Kübra Özkan Karacaer, Semir Paşa, Fatma Sılan

**Affiliations:** 1 Çanakkale Onsekiz Mart University Faculty of Medicine, Department of Gynecology and Obstetrics, Çanakkale, Turkey; 2 Dokuz Eylül University Faculty of Medicine, Department of Gynecology and Obstetrics, İzmir, Turkey; 3 Mardin Artuklu University Faculty of Health Sciences, Mardin, Turkey; 4 Çanakkale Onsekiz Mart University Faculty of Medicine, Department of Medical Genetic, Çanakkale, Turkey

**Keywords:** Primary ovarian insufficiency, inflammation, hemogram

## Abstract

**Objective:**

In most of primary ovarian insufficiency (POI) cases, etiologic factors have not been fully elucidated. Recent studies have revealed that inflammatory agents play an important role in the etiopathogenesis of POI. Therefore, the aim of this study was to investigate the role of inflammatory markers of hemogram parameters in POI.

**Materials and Methods:**

The study compared 47 healthy women and 47 women diagnosed as having POI retrospectively by scanning electronic and written recording systems. Complete blood counts, day-3 hormone profiles levels of all subjects were analyzed. The neutrophil-lymphocyte ratio (NLR), red cell distribution width (RDW), platelet ratio (RPR), platelet lymphocyte ratio (PLR), and mean platelet volume (MPV) mean platelet lymphocyte ratio (MPLR) were calculated from the complete blood count parameters.

**Results:**

White blood cell and MPV values, platelet, and lymphocyte counts were significantly higher in the POI patients (p<0.001, p=0.042, p=0.038, p=0.049, respectively), RPR was significantly lower than the control group (p=0.011), but there were no significant differences in hemoglobin, RDW, NLR, PLR, and MPLR (p=0.454, p=0.057, p=0.635, p=0.780, p=0.126, respectively). The neutrophil count of the study group was higher than in the control group (p=0.057). Bivariate correlation analyses showed no correlations between blood parameters and hormone levels. The area under the receiver operating characteristic curve for RPR in POI was 0.652, with a threshold value 0.053, sensitivity=63% and specificity=63.

**Conclusion:**

Inflammatory markers of hemogram detected higher in patients with POI then control subjects.

**PRECIS:** Inflammatory markers of hemogram detected higher in patients with POI then control subjects.

## Introduction

Primary ovarian insufficiency (POI), also known as premature ovarian failure prior to 2008, is defined as the presence of reduced ovarian functions in women younger than 40 years, characterized by oligo- or amenorrhea, sub- or infertility, loss of residual follicles in the gonads, low estradiol levels, and high (menopausal) follicle-stimulating hormone (FSH) levels^(1,2)^. The risk of POI in women before the age of 40 is 1%^(3)^. POI is seen in one ten thousand^th^ in the 18-25 age group, one thousand^th^ in the 25-30 age group, and one percent in the 35-40 age group. Fifteen percent of patients with POI are familial, suggesting an underlying genetic factor^(4,5)^.

In the case of oligo- or amenorrhea (primary or secondary) for 4-6 months, the presence of hypergonadotropic hypogonadism in any woman younger than 40 years is sufficient for the diagnosis of POI^(6)^. However, symptoms of hypoestrogenism, the decrease in ovarian functions, and pace of events may affect the clinical picture. Thus, clinical manifestations of POI are diverse, including underlying etiologic factors, and the clinical manifestations of hypoestrogenism, such as hot flashes, dyspareunia, sleep disorders, decreased libido or vaginal dryness. Therefore, clinical manifestations of POI are diverse, including clinical signs of underlying etiologic factors and symptoms of hypoestrogenism such as hot flashes, dyspareunia, sleep disorders, decreased libido or vaginal dryness^(7,8)^.

A number of reasons for the etiopathogenesis of POI have been elucidated. Some of them are genetic anomalies, metabolic diseases, autoimmunity, iatrogenic events such as chemotherapy, infections, and environmental factors. However, in most cases the etiologic factor cannot be known and therefore these cases are called spontaneous or idiopathic^(1)^. Recent studies have revealed that inflammatory agents play an important role in the etiopathogenesis of POI. In ovarian biopsies of patients with POI, it was shown that there are inflammatory cells associated with lymphocytic infiltration and other immune responses^(9,10,11,12,13). ^Inflamm-aging, defined as inflammation caused by aging, and inflammatory cells reacting at ovaries as a result of autoimmunity play an important role in idiopathic and unknown cases^(14,15)^. It has also been demonstrated in studies that estrogen depletion leads to pro-inflammatory processes secondary to an increase in proinflammatory markers, and oral estrogen treatment leads to a reduction in inflammatory marker concentrations^(16,17,18,19,20)^. Inflammation, which plays a role in the etiopathogenesis of POI, and increased inflammatory markers triggered by decreased estrogen after POI development enter a cycle that triggers each other.

The aim of this study was to reveal the pathophysiologic role of inflammation and inflammatory markers in POI by comparing inflammatory markers including neutrophils, lymphocytes, and the values of hemoglobin (HGB), white blood cell (WBC), mean platelet volume (MPV), red cell distribution width (RDW), neutrophil lymphocyte ratio (NLR), RDW platelet ratio (RPR), platelet lymphocyte ratio (PLR), mean platelet lymphocyte ratio (MPLR) with a control group, to determine whether these markers could be used in the diagnosis and to investigate relationships between these markers and hormones such as FSH and luteinizing hormone (LH).

## Materials and Methods

This study was conducted as a retrospective and comparative cross sectional study at Çanakkale Onsekiz Mart University Hospital between January 2016 and December 2018 and Çanakkale State Hospital between January 2016 and December 2017. The study group comprised 47 patients with POI who were admitted to these clinics, and the control group consisted of 47 healthy females. All procedures performed in human studies were conducted in accordance with the ethical standards of the institutional and/or national research committee and the ethical standards of the 1964 Helsinki Declaration. The study was approved by Çanakkale Onsekiz Mart University Local Ethics Committee (approval number: 02.05.2018/09).

Patients aged between 20 and 40 years who were diagnosed as having POI were included in the study group. Women aged <40 years with elevated FSH levels >25 mIU/mL and accompanying 4-6 months of amenorrhea or oligomenorrhea periods, as identified in european society of human reproduction and embryology guideline, were considered as having POI^(6)^. Exclusion criteria of the study group were polycystic ovary syndrome, pregnancy, chronic medical disease, hyperprolactinemia, presence of hypothalamic or pituitary disease, hyperthyroidism, chemotherapy, and pelvic surgery prior to study and abnormal karyotype analysis. The inclusion criteria for healthy individuals in the control group were in the age range of 20-40 years, regular menstrual cycles, no use of drugs including contraceptive hormone therapies, and no known chronic disease.

From all of the participants, blood samples taken after 8-10 hours of fasting were collected in a tube with ethylenediamine tetraacetic acid (EDTA) and in a gel tube without anticoagulant. Complete blood counts in the tubes with EDTA were analyzed using an auto hematology analyzer. In this study, we evaluated the analytical performance of automated cellular analyzers for BF analysis: the UniCel DxH 800 (Beckman Coulter, Brea, CA, USA). Plasma levels of FSH, LH, prolactin, and thyroid-stimulating hormone in the gel tubes were measured by electrochemiluminescence immunoassay on a Cobas 6000 analyzer (Roche Diagnostics Germany) after centrifuging and separating their serum. NLR, RPR, PLR, and MPLR were calculated from the complete blood count parameters.

### Statistical Analysis

Statistical analysis was performed using the Statistical Package for the Social Science (SPSS) statistics 20 software. The data were reported as the mean ± SD and median (minimum-maximum). The Shapiro-Wilk tests, skewness and kurtosis values, histograms, and detrended normal q-q plots were used for normality analyses of parameters. Student’s t-test was used for comparisons between groups of parameters with normal distribution, and the Mann-Whitney U test was used for parameters with non-normal distribution. According to parameter suitability Pearson’s (for normal distribution) or Spearman’s correlation tests were used for correlation analyses. P<0.05 was considered as statistically significant.

## Results

The average and median age of patients with POI and the control group were 31.6±7.05 and 35 (range, 20-40) years and 30.3±5.38 and 31 (range, 20-39) years. The baseline characteristics and hormone levels of both groups are shown in Table 1.

The mean levels (with standard deviations) and median levels (with minimum and maximum) of blood parameters of 47 patients with POI and 47 healthy individuals are depicted in Table 2. When the parameters in the complete blood count were analyzed, it was found that values of WBC and MPV, platelet and lymphocyte counts were significantly higher in the POI patients (p<0.001, p=0.042, p=0.038, p=0.049, respectively), RPR was significantly lower than the control group (p=0.011), but there were no significant differences in HGB, RDW, NLR, PLR, and MPLR (p=0.454, p=0.057, p=0.635, p=0.780, p=0.126, respectively). The neutrophil count of the study group was higher than in the control group. However, even though there was not a significant relationship, the p value was very near to 0.05 (p=0.057).

Bivariate correlation analyses were conducted to determine relationships between the hormone levels (FSH and LH) and parameters of complete blood count in patients with POI (Table 3). However, no correlations were found between blood parameters and hormone levels in patients with POI.

**Figure 1:**
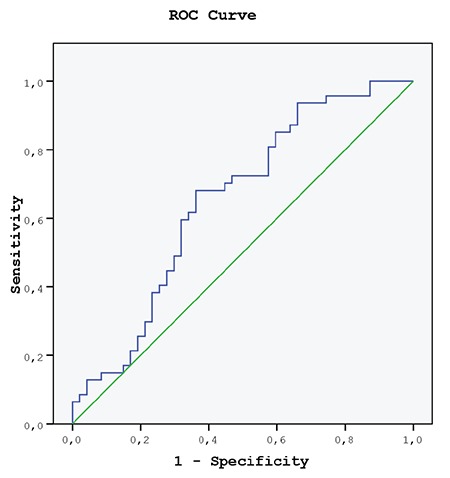
Receiver operating characteristic curve of platelet ratio to predict cases with primary ovarian insufficiency ROC: Receiver operating characteristic

We also performed receiver operating characteristic (ROC) curve analysis for RPR in the POI group. The area under the ROC curve for RPR in POI was 0.652 (Figure 1), with a threshold value 0.053. It discriminated patients with POI lower than 0.053 with a sensitivity=63% and specificity=63% (95% CI: 0.540-0.764; p=0.011).

## Discussion

The etiology is not known in 90% of POI cases and it is thought that autoimmunity and inflammation may play an important role in ovarian dysfunction in most of these unknown cases. In recent years, some studies have been published on the increasing importance of inflammatory agents in ovarian diseases. Interests in this issue have been raised by the detection of xanthogranulomatous inflammation in a case with POI by Singh et al.^(21)^. In an experimental study on mice by Altuntas et al.^(22)^, it was demonstrated that the targeting action of inhibin-alpha on autoimmunity was initiated by CD4 T helper cells, by stimulating B cells. Said et al.^(23)^ also found that in primary ovarian failure induced by ionizing radiation and resveratrol, the expression of proinflammatory factors was increased and anti-inflammatory factors were inhibited. Tung et al.^(24)^ showed that in loss of immune regulation in a mouse model, B lymphocytes stimulated by proinflammatory T lymphocytes and autoantibodies against oocyte antigens played important roles in the pathogenesis of POI. Huang et al.^(14)^ claimed in their review article that increased inflammatory cytokines and decreased anti-inflammatory cytokines played a crucial role in aging-related inflammation and POI. In addition, a decrease in the body’s estrogen concentration during POI can lead to the activation of the pro-inflammatory pathway and B cells, tumor necrosis factor-α, interferon-γ, interleukin 1 and natural killer cells involved in this pathway^(16)^.

Randomized controlled studies investigating the role of inflammatory markers in etiopathogenesis of POI is not sufficiently available in the literature. The first study on this subject was conducted by Miyake et al.^(25)^. In this study, subtypes of lymphocytes and autoantibodies of peripheral blood lymphocytes were investigated in 20 patients with POI. They found that the total lymphocyte count and CD4 T lymphocyte count were increased and CD8 T lymphocytes count was decreased in patients with POI compared with women in the same age group. However, these differences were not significant. Although lymphocyte subgroups were not investigated in our study, the lymphocyte count was significantly increased in the POI patients. In recent years, there have been studies showing that the lymphocyte count increased significantly in patients with POI; however, there are also studies in which no significant differences were found. In a study conducted by Yıldırım et al.^(26)^ on investigating inflammatory markers including 43 patients in the study group and 41 women in the control group, they showed that lymphocyte counts were significantly increased in the study group, but there were no significant differences in neutrophil counts and values of WBC and MPV. In a study conducted by Sanverdi et al.^(20)^ on 96 patients POI and 110 healthy women, although the lymphocyte count was significantly increased and neutrophil count was significantly decreased, no significant changes were observed in parameters including MPV, RDW, and platelet count. However, İlhan et al.^(36)^ and Akdemir et al.^(27)^ found no significant differences between neutrophil and lymphocyte counts in their studies on complete blood counts in patients with POI^(20)^. In our results, values of WBCs and MPV, platelets, and lymphocyte counts were significantly increased in patients with POI, but there were no significant changes in values of HBG, RDW, and neutrophil count compared with the control group. While the above-mentioned studies showed no significant correlations between hormone levels and inflammatory parameters, there were also no significant correlations between them in our study.

The NLR, RPR, PLR, and MPLR have been reported as laboratory markers for inflammation and diseases related to inflammatory processes^(28)^. This NLR is a method that is frequently used in recent years to determine the degree of inflammation because it is easy to measure, frequent, and cheap^(29,30,31,32,33,34,35,36)^. In the literature, the predictive value of NLR in diabetes mellitus, thyroid dysfunction, essential hypertension, renal disease, heart valve disease, autoimmune diseases, and malignancies including ovarian, lung, renal, and colorectal cancers has been investigated^(29,30,31,32,33,34,35,36,37,38,39,40)^. The PLR has also been found to be an independent risk factor for survival in patients with malignancies such as pancreatic and colorectal cancer^(37,38,39,40)^. The RPR has resulted in a valuable new laboratory test for predicting mortality in acute pancreatitis, hepatic fibrosis, and cirrhosis^(41,42)^. The PLR was also found to be an independent risk factor for survival in patients with malignancies such as pancreatic and colorectal cancer. The predictive value of NLR in POI and whether it can be used in the diagnosis of POI has been a frequent research topic in recent studies. İlhan et al.^(36)^ used NLR, PLR, and RPR as diagnostic markers in patients with POI and they found that only NLR showed a statistically significant difference between the groups. ROC analysis showed that NLR could be a marker for the diagnosis of POI. In this study, NLR was found to be significantly increased in patients with POI, whereas Sanverdi et al.^(20)^ and Yildirim et al.^(26)^ found that NLR was significantly decreased in women with POI. In the study conducted by Akdemir et al.^(27)^, there was no significant difference in NLR rates between the groups, as we found. Sanverdi et al.^(20)^ also found that MPLR was significantly higher in the POI group and MPLR might be a marker for the diagnosis of POI. When the results of this literature and our results are evaluated together, it can be concluded that the use of NLR in the early diagnosis of POI is not correct, but according to our ROC analysis, RPR can be used for the diagnosis of POI.

## Conclusion

In conclusion, inflammatory markers of hemogram were detected higher in patients with POI than in control subjects. Even though the role of inflammatory cytokines in the etiopathogenesis of POI has been elucidated in experimental studies, it has not been adequately demonstrated in clinical studies. More studies with more patient groups are needed to reveal the role of inflammation and inflammatory markers of hemogram in the pathogenesis of POI.

## Figures and Tables

**Table 1:**
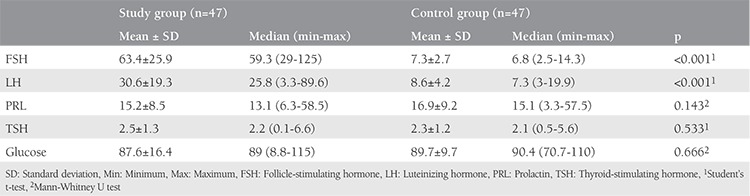
Baseline characteristics and hormone levels of the primary ovarian insufficiency patients and the control group

**Table 2:**
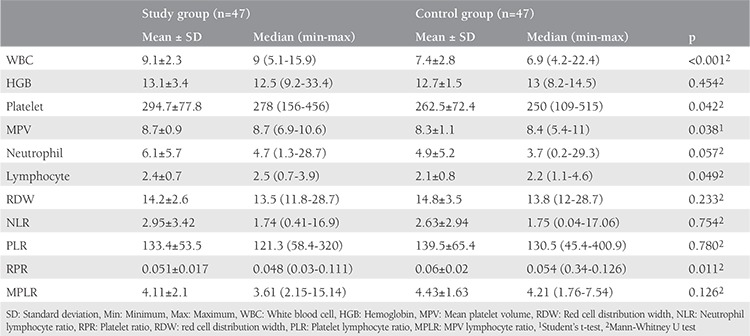
Blood parameters of the primary ovarian insufficiency patients and the control group

**Table 3:**
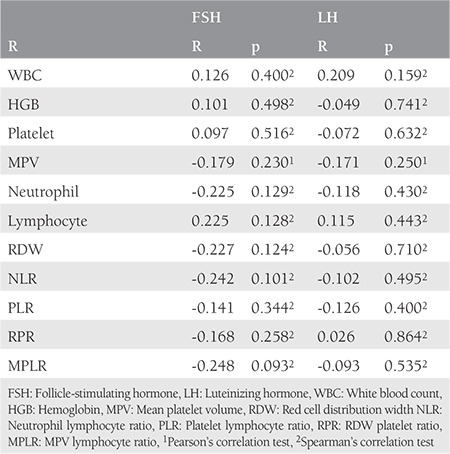
Correlations between follicle-stimulating hormone, luteinizing hormone, and blood parameters

## References

[1] Rudnicka E, Kruszewska J, Klicka K, Kowalczyk J, Grymowicz M, Skórska J, et al (2018). Premature ovarian insufficiency - aetiopathology, epidemiology, and diagnostic evaluation. Prz Menopauzaln..

[2] Bukulmez O (2020). Definitions and Relevance: Diminished Ovarian Reserve, Poor Ovarian Response, Advanced Reproductive Age, and Premature Ovarian Insufficiency. Diminished Ovarian Reserve and Assisted Reproductive Technologies: Springer.

[3] Coulam CB, Adamson SC, Annegers JF (1986). Incidence of premature ovarian failure. Obstet Gynecol.

[4] Franic-Ivanisevic M, Franic D, Ivovic M, Tancic-Gajic M, Marina L, Barac M, et al (2016). Genetic Etiology of Primary Premature Ovarian Insufficiency. Acta Clin Croat.

[5] Cramer DW, Xu H (1996). Predicting age at menopause. Maturitas..

[6] Webber L, Davies M, Anderson R, Bartlett J, Braat D, Cartwright B, et al (2016). ESHRE Guideline: management of women with premature ovarian insufficiency. Hum Reprod.

[7] Torrealday S, Kodaman P, Pal L (2017). Premature Ovarian Insufficiency - an update on recent advances in understanding and management. F1000Res.

[8] Rebar RW (2009). Premature ovarian failure. Obstet Gynecol..

[9] Sen A, Kushnir VA, Barad DH, Gleicher NSen A, Kushnir VA, Barad DH, Gleicher N (2014). Endocrine Autoimmune Diseases and Female Infertility. Nat Rev Endocrinol.

[10] Sammaritano LR (2012). Menopause in Patients With Autoimmune Diseases. Autoimmun Rev.

[11] Petríková J, Lazurova I (2012). Ovarian failure and polycystic ovary syndrome. Autoimmunity Reviews.

[12] Carp HJA, Selmi C, Shoenfeld Y (2012). The Autoimmune Bases of Infertility and Pregnancy Loss. J Autoimmun.

[13] Reato G, Morlin L, Chen S, Furmaniak J, Smith BR, Masiero S, et al (2011). Premature Ovarian Failure in Patients With Autoimmune Addison’s Disease: Clinical, Genetic, and Immunological Evaluation. J Clin Endocrinol Metab..

[14] Huang Y, Hu C, Ye H, Luo R, Fu X, Li X, et al (2019). Inflamm-Aging: A New Mechanism Affecting Premature Ovarian Insufficiency. Journal of Immunology Rsearch..

[15] Sharif K, Watad A, Bridgewood C, Kanduc D, Amital H, Shoenfeld Y (2019). Insights Into the Autoimmune Aspect of Premature Ovarian Insufficiency. Best Pract Res Clin Endocrinol Metab.

[16] Straub RH (2007). The Complex Role of Estrogens in Inflammation. Endocr Rev.

[17] Fröhlich M, Mühlberger N, Hanke H, Imhof A, Döring A, Pepys MB, et al (2003). Markers of inflammation in women on different hormone replacement therapies. Annals of Medicine.

[18] Lacut K, Oger E, Le Gal G, Blouch M-T, Abgrall J-F, Kerlan V, et al (2003). Differential Effects of Oral and Transdermal Postmenopausal Estrogen Replacement Therapies on C-reactive Protein. Thrombosis and haemostasis.

[19] Silvestri A, Gebara O, Vitale C, Wajngarten M, Leonardo F, Ramires JA, et al (2003). Increased Levels of C-reactive Protein After Oral Hormone Replacement Therapy May Not Be Related to an Increased Inflammatory Response. Circulation.

[20] Sanverdi I, Kilicci C, Cogendez E, Abide Yayla C, Ozkaya E (2018). Utility of Complete Blood Count Parameters to Detect Premature Ovarian Insufficiency in Cases with Oligomenorrhea/Amenorrhea. J Clin Lab Anal.

[21] Singh N, Dadhwal V, Sharma KA, Mittal S (2009). Xanthogranulomatous Inflammation: A Rare Cause of Premature Ovarian Failure. Arch Gynecol Obstet.

[22] Altuntas CZ, Johnson JM, Tuohy VK (2006). Autoimmune Targeted Disruption Of The Pituitary-Ovarian Axis Causes Premature Ovarian Failure. J Immunol.

[23] Said RS, El-Demerdash E, Nada AS, Kamal MM (2016). Resveratrol Inhibits Inflammatory Signaling Implicated in Ionizing Radiation-Induced Premature Ovarian Failure Through Antagonistic Crosstalk Between Silencing Information Regulator 1 (SIRT1) and poly (ADP-ribose) Polymerase 1 (PARP-1). Biochem Pharmacol.

[24] Tung KS, Garza KM, Lou Y, Bagavant H (2001). Autoimmune Ovarian Disease: Mechanism of Induction and Prevention. J Soc Gynecol Investig.

[25] Miyake T, Sato Y, Takeuchi S (1987). Implications of Circulating Autoantibodies and Peripheral Blood Lymphocyte Subsets for the Genesis of Premature Ovarian Failure. J Reprod Immunol.

[26] Yıldırım G, Tokmak A, Kokanal MK, Sarkaya E, Züngün C, Inal HA, et al (2015). Association Between Some Inflammatory Markers and Primary Ovarian Insufficiency. Menopause.

[27] Akdemir N, Bostanci MS, Tozlu F, Özden S, Ünal O, Cevrioglu AS (2016). Neutrophil/lymphocyte ratio as new-inflammatory biomarkers Primary ovarian insufficiency (POI) patients. Journal of The Turkish German Gynecological Association.

[28] Toktas O, Aslan M (2017). Mean platelet volume, red cell distribution width, neutrophil to lymphocyte ratio and platelet to lymphocyte ratio in the diagnosis acute appendicitis. Eastern Journal Medicine.

[29] Yamanaka T, Matsumoto S, Teramukai S, Ishiwata R, Nagai Y, Fukushima M (2007). The Baseline Ratio of Neutrophils to Lymphocytes is Associated With Patient Prognosis in Advanced Gastric Cancer. Oncology.

[30] Cho H, Hur HW, Kim SW, Kim SH, Kim JH, Kim YT, et al (2009). Pretreatment Neutrophil to Lymphocyte Ratio is Elevated in Epithelial Ovarian Cancer and Predicts Survival After Treatment. Cancer Immunol, Immunother.

[31] Azab B, Jaglall N, Atallah JP, Lamet A, Raja-Surya V, Farah B, et al (2011). Neutrophil-lymphocyte Ratio as a Predictor of Adverse Outcomes of Acute Pancreatitis. Pancreatology.

[32] Guthrie GJ, Charles KA, Roxburgh CS, Horgan PG, McMillan DC, Clarke SJ (2013). The Systemic Inflammation-Based Neutrophil– Lymphocyte Ratio: Experience in Patients With Cancer. Crit Rev Oncol Hematol..

[33] Stotz M, Gerger A, Eisner F, Szkandera J, Loibner H, Ress A, et al (2013). Increased Neutrophil-Lymphocyte Ratio Is a Poor Prognostic Factor in Patients With Primary Operable and Inoperable Pancreatic Cancer. Br J Cancer.

[34] Zheng Y-B, Zhao W, Liu B, Lu L-G, He X, Huang J-W, et al (2013). The Blood Neutrophil-To-Lymphocyte Ratio Predicts Survival in Patients With Advanced Hepatocellular Carcinoma Receiving Sorafenib. Asian Pac J Cancer Prev.

[35] Tanoglu A, Karagoz E (2014). Predictive Role of the Neutrophil-To-Lymphocyte Ratio in Patients With Advanced Hepatocellular Carcinoma Receiving Sorafenib. Asian Pac J Cancer Prev.

[36] İlhan M, İlhan G, Gök AFK, Bademler S, Verit Atmaca F, Ertekin C (2016). Evaluation of Neutrophil–Lymphocyte Ratio, Platelet–Lymphocyte Ratio and Red Blood Cell Distribution Width–Platelet Ratio as Early Predictor of Acute Pancreatitis in Pregnancy. J Matern Fetal Neonatal Med.

[37] Betterle C, Volpato M, Smith BR, Furmaniak J, Chen S, Greggio NA, et al (1997). I. Adrenal Cortex and Steroid 21-Hydroxylase Autoantibodies in Adult Patients With Organ-Specific Autoimmune Diseases: Markers of Low Progression to Clinical Addison’s Disease. J Clin Endocrinol Metab.

[38] van Der Voort D, van Der Weijer P, Barentsen R (2003). Early Menopause: Increased Fracture Risk at Older Age. Osteoporosis Int.

[39] Schmidt PJ, Cardoso GM, Ross JL, Haq N, Rubinow DR, Bondy CA (2006). Shyness, Social Anxiety, and Impaired Self-Esteem in Turner Syndrome and Premature Ovarian Failure. Jama.

[40] Shuster LT, Rhodes DJ, Gostout BS, Grossardt BR, Rocca WA (2010). Premature Menopause or Early Menopause: Long-Term Health Consequences. Maturitas..

[41] Chen B, Ye B, Zhang J, Ying L, Chen Y (2013). RDW to Platelet Ratio: A Novel Noninvasive Index for Predicting Hepatic Fibrosis and Cirrhosis in Chronic Hepatitis B. PLoS one..

[42] Çetinkaya E, Şenol K, Saylam B, Tez M (2014). Red cell distribution width to platelet ratio: New and promising prognostic marker in acute pancreatitis. World J Gastroenterol.

